# Methylenetetrahydrofolate reductase polymorphism (MTHFR C677T) and headache in children: a retrospective study from a tertiary level outpatient service

**DOI:** 10.1186/s13052-018-0546-1

**Published:** 2018-08-31

**Authors:** A. Orsini, I. Sammartino, A. Valetto, V. Bertini, P. Marchese, A. Bonuccelli, D. G. Peroni

**Affiliations:** 1 Pediatric Neurology, Pediatric Department, Azienda Ospedaliera Universitaria Pisana, Via Roma 57, 56100 Pisa, Italy; 2 Cytogenetics Unit, Medicine of Laboratory Department, Azienda Ospedaliera Universitaria Pisana, Pisa, 56100 Italy

**Keywords:** Children migraine, MTHFR C677T, Hyperhomocysteinemia, Migraine with aura, Migraine, MTHFR

## Abstract

**Background:**

In adult studies the MTHFR C677T polymorphism has been associated with an increased risk of migraine, but little research has been done in this area in children.

**Methods:**

A retrospective study of children referred with headache to a tertiary level Paediatric Neurology Service between 2008 and 2012. This study included only patients who had been genotyped for the MTHFR C677T polymorphism. An evaluation of homocysteine serum levels was necessary to exclude other types of migraine.

**Conclusion:**

Compared with the wild-type genotype, the T/T genotype was associated with an increased risk of any type of migraine, though the statistical significance was greatest in migraine with aura. The homocysteine serum levels were significantly higher in migraine with aura compared to migraine without aura. In a pediatric population MTHFR T/T homozygosity influences susceptibility to migraine.

## Background

The prevalence of migraine in the pediatric population ranges from 3.3 to 21.4% and increases from childhood to adolescence [1]. It is frequently accompanied by comorbid conditions and can have a significant negative impact on children’s quality of life and school performance [1].

At present, without validated biochemical investigations, migraine remains a clinical diagnosis supported by the diagnostic criteria outlined by the International Headache Society (IHS) [2]. The IHS have comprehensively classified tension-type headache (TTH) and migraine, with migraine divided in turn into two main subtypes; migraine without aura (MOA) and migraine with aura (MA) [2].

Migraine is a multifactorial disease and it is suspected that a significant part of the etiology is genetic with strong familial aggregation. It is likely that both migraine subtypes have some genetic determinants in common, although different modifying factors, including genetic and lifestyle triggers, may contribute to the variable clinical phenotype [3]. The genes involved in regulating the vascular system, especially the MTHFR gene, have been linked to the pathogenesis of both migraine subtypes [4–8].

The human MTHFR gene mapped to chromosome 1p36.3 catalyses the conversion of 5, 10-methylenetetrahydrofolate (5, 10-CH2-THF) to 5-methyltetrahydrofolate (5-CH3-THF), the principal circulatory form of folate and a cofactor for methylation of homocysteine to methionine [7–9].

A common MTHFR gene variant (C677T) alters the amino acid sequence, substituting alanine (Ala) with valine (Val). Individuals with the Val residue may exhibit significantly reduced MTHFR enzyme activity whereby, compared to baseline levels, the mean activity is 65% in the Ala/Val heterozygote and 30% in the Val/Val homozygous state [10].The latter enzymatic form can lead to mild elevation in plasma homocysteine levels particularly if dietary folate intake is low [3, 11].

The C677T polymorphism of the MTHFR gene has been associated with a range of diseases such as stroke, coronary artery disease and psychiatric disorders. Previous research investigating the association between this polymorphism and migraine has produced conflicting results [4–8]. However, there have been several studies and meta-analyses published that demonstrate an association specifically between increased risk of MA in adult populations and the MTHFR gene Т/Т polymorphism [6, 7]. Previous studies performed in pediatric populations have not always included both subtypes of migraine and have been limited by their small sample size (Table 1) [4, 5, 8, 9, 12]. Further study in larger populations has been recommended to support their findings of a potential relationship between MTHFR polymorphisms, high homocysteine serum levels and migraine [4, 5, 8, 9, 12].

**Table 1:** Summary of cases collected in the study

	T/T	C/T	C/C	TOTAL	Mean Homocysteine μMol/L	onset T/T	onset C/C
MA	34 (47.9%)	22	15	71	7.85	11.73 yr	11.87 yr
MOA	43 (28.8%)	71	35	149	5.82	8.55 yr	8.86 yr
MA + MOA	77(35%)	93	50	220	6.48	9.95 yr	9.76 yr
TTH	12 (13.5%)	45	47	104	7.23	11.5 yr	9.85 yr
HF	20 (19.4%)	49	34	103			
TOTAL	109 (25.7%)	187	131	427	6.66	10.16 yr	9.80 yr

## Methods

The aim of this study was to evaluate any association between MTHFR T/T polymorphism, high homocysteine serum levels and migraine, in a large pediatric population.

We retrospectively evaluated all pediatric patients referred with headache to our tertiary level, neuropediatric service between 2008 and 2017. From these, we selected children who were diagnosed with migraine. Diagnosis was made by two independent pediatric neurologists using the IHS second edition criteria, which considers family and personal history, clinical and neurological examination. Secondary causes of headache were excluded with relevant investigations. Patients with migraine were only included in the study if they had been investigated for MTHFR C677T polymorphism and serum homocysteine levels at their first examination for diagnostic purposes, with the aim of trying to exclude secondary causes of migraine. As controls, we selected patients investigated with MTHFR C677T polymorphism and serum homocysteine levels for different reasons, in particular, for the presence in family members of the MTHFR C677T polymorphism, for positive familiar anamnesis of thrombosis, for arthritis or syncope episodes without headache comorbidity. We considered as normal range of homocysteine levels between 4.3 and 11.1 μmol/L according to our laboratory.

The MTHFR C677T polymorphism was genotyped using a Polymerase Chain Reaction (PCR) to amplify the desired regions of the gene MTHFR at 677. The amplified PCR products were subjected to restriction enzyme digestion. Digestion produced a 175 and 23 base pair (bp) fragments for TT condition (homozygous polymorphic) and a 198,175 and 23 bp fragments for CT condition (heterozygous polymorphic). An undigested product length of 198 bp was retained by the wild types.

We used the old International Classification of Headache Disorders (ICHD) criteria because most of the patients were gathered when the old ICHD criteria were the most recent.

All of the patient data were collected from the hospital database, using information gathered during routine clinical practice. A standardized data extraction form was created for this purpose, including information about gender, polymorphism of MTHFR gene, headache type and homocysteine level.

The statistical analysis and graphs were conducted using JMP 10 software. Gaussian variables were expressed as mean ± standard deviation, while median results were reported for any data that was not normally distributed. Normality of distributions of homocysteine was assessed using relative logarithm in base 10. The other parameters were already in a normal distribution. A t-test was performed to analyze differences between continuous variables and the Kruskal-Wallis testto assess for differences between nominal variables.

## Results

A total of 427 patients met the inclusion criteria and were included for analysis, of whom261(61.12%) were female and 166 (38.88%) male. The age of onset was between 2 and 18 years, with an average age of 9.90 ± 3.46 years. 149 (34.9%) had MOA, consisting of 95 females and 54 males with an average age of 8.79 ± 3.11 years. Seventy-one (16.62%) were diagnosed with MA, consisting of 43 females and 28 males with an average age of 12.16 ± 3.28 years. 104 (32.1%) were diagnosed with tension-type headache (TTH), consisting of 60 females and 44 males with an average age of 9.97 ± 3.33 years. 103 (24.12%) patients, headache free (HF), were evaluated as control, consisting in 62 females and 41 males with an average age of 11.61 ± 4.04 years (Table 1). Among the total of our population 131 (30.7%) patients had the C/C genotype, 187 (43.8%) patients had the C/T genotype and 109 (25.5%) had the T/T genotype.

A significantly higher proportion of patients diagnosed with MA (*p* < 0.0001) were found to have the T/T allele (n 34, 47.9%) than the MOA patients (n 43, 28.8) and HF (*p* = 0.0019) as described in the literature in pediatric and adult population [4, 5, 7, 12] (Tables 1 and 2). No differences were found between the TTH group and the HF (*p* = 0.19).

**Table 2:** Previous studies that analyzed the relationship between MTHFR polymorphisms migraine compared to ours

	C677T MTHFR Polymorphism TT genotype in children with migraine	C677T MTHFR Polymorphism TT genotype in general pediatric population	Age in years
Orsini et al. (our study).	77/220 (35%)	–	9.83 (3–17)
Hamza Alsayouf et al. (J.child neurology, 2011)	11/41 (26.8%)	–	15 (1 day-20 yrs)
Bottini et al., (Cephalalgia. 2006)	12/45 (26.7%)	9/66 (13.6%) (age 15–41)	11.6 (5–17)
Di Rosa G et al., Headache. 2007	11/16 (68%)	–	12 (8–18)
Ferrara M [12], Hematology. 2012	15/42 (35.7%)	11.9% (age 9 to 15)	8.9 (7.5–14.5).
Herak DC, Pediatrics. 2009	6/35 (17.1%)	10/112 (8.9%) (age < 18 years)	12 (6–17)

Regarding the genotype, a significant difference was found between MA and HF (*p* = 0.0006; OR: 3, 81 95%CI: 1.94–7, 49), which directly relates the genotype T/T more frequently with MA; another, but less significant, difference was found between genotype MOA and HF patients (*p* = 0.041; OR: 1.68 95%CI: 0.92–3.01), which directly relates the genotype T/T more frequently to MOA than HF patients. These results confirm the direct association of T/T genotype firstly with MA and to a lesser extent with MOA.

Across all patients diagnosed with a migraine (MA + MOA) the frequency of the T/T genotype was 35% (77/220) compared to 8–13% in the general pediatric and adult population [4–7, 12] (Table 1).

Homocysteine blood levels were significantly higher in participants with MA (average value 7.59 ± 4.61 μMol/L) than in MOA group (average value 5.85 ± 2.62μMol/L) (*p* = 0.023). (Table 1).

We also divided the population into 2 subgroups: patients with migraine (220/427) and patients without migraine, and we reported a significant relation with the T/T genotype and migraine group (*p* < 0.0001). We then stratified the population in two other subgroups: patients with MA and patients without MA, in order to understand selectively if the aura was related to the genotype, and actually the T/T genotype is more related to MA than C/T (*p* < 0.0001) or C/C (*p* = 0.0001). These results are confirmed by observing the significant correlation between homocysteine levels and the presence of MA (*p* = 0.0183), and the absence of correlation with the migraine itself (*p* = 0.61).

Patients with the T/T genotype and MA had an average age of reported symptom onset of 11.73 ± 3.66 years compared with an average age of 12.56 ± 2.88 in C/T and C/C genotype. In patients diagnosed with MOA, the average age of reported symptom onset was 8.55 ± 3.19 years for those with the T/T genotype and 8.90 ± 3.09 years for those with either the C/T or C/C genotype.

**Fig. 1:**
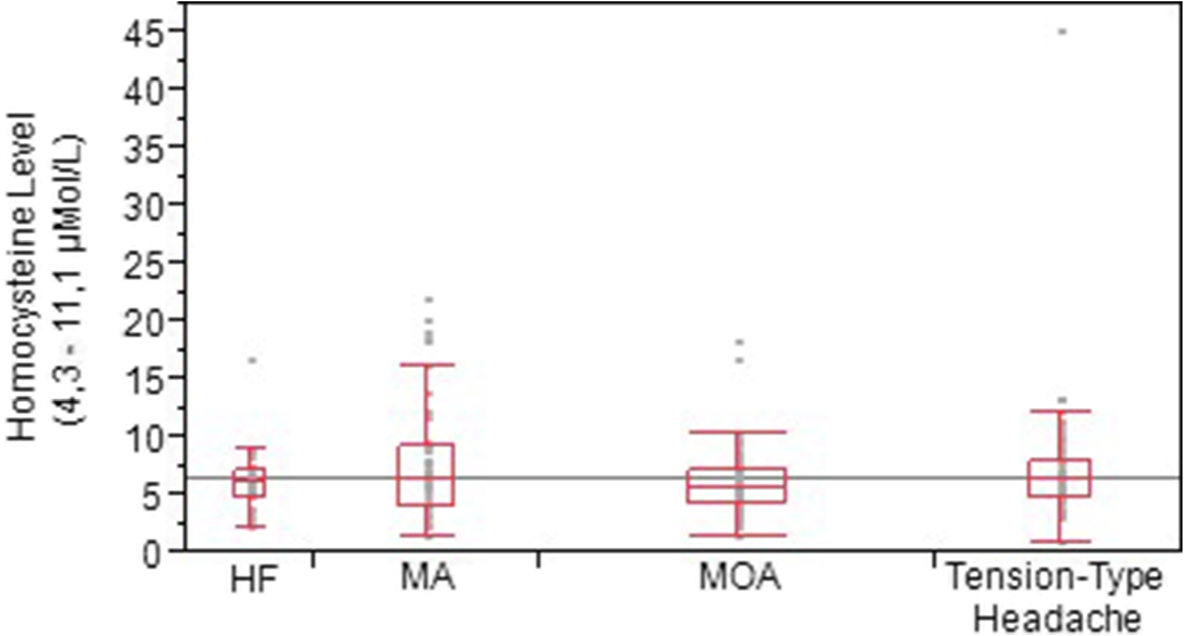
Distribution of MTHFR polymorphism among the groups; Headache Free (HF), Migraine with Aura (MA), Tension-type headache (TTH), Migraine without aura (MOA)

Within the study population, there was a statistically significant difference between the headache type and the genetic polymorphism (*p* < 0.0001):the T/T genotype was found in 47.8% of patients with MA, in 28.9% of patients with MOA and 11% of patients with TTH. (Fig. 1).To understand if the problem was the mutated allele itself or its effect on human metabolism, the population was analyzed in two groups based on homocysteine levels. Lower levels of homocysteine were associated more with TTH and HF patients compared to MOA and MA (*p* < 0.0001).

**Fig. 2:**
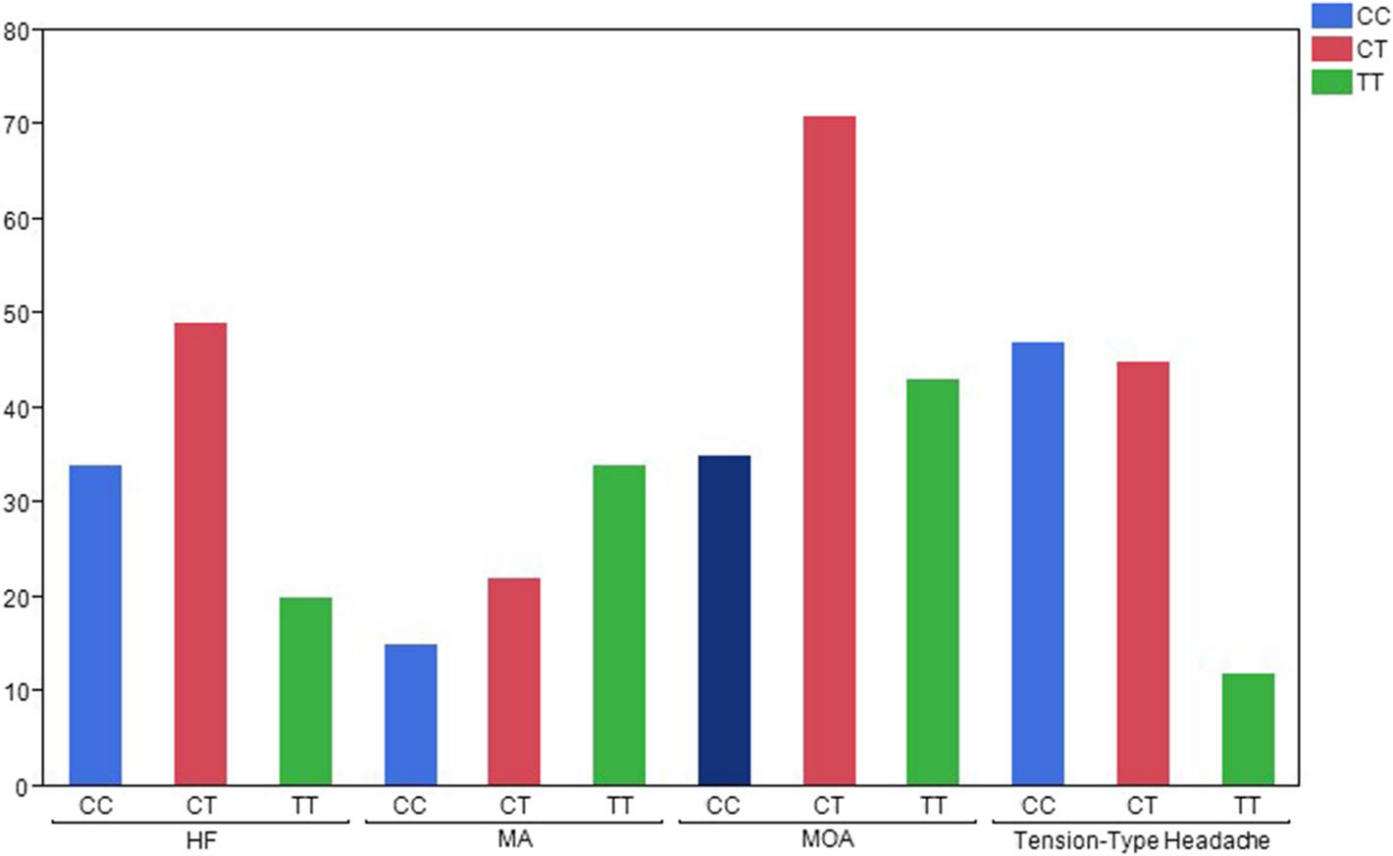
Distribution of Homocysteine levels among the groups; Headache Free (HF), Migraine with Aura (MA), Tension-type headache (TTH), Migraine without aura (MOA)

Making a comparison between the homocysteine value and the type of headache, a statistically significant difference is found (*p* = 0.0204), in particular, among the patients of the MA and MOA groups (*p* = 0.023). This result suggests that a higher value of homocysteine is more related toa MA rather than MOA. That result is also demonstrated in analyzing according to sex: the same statistically significant difference is in fact observed in the female group (*p* = 0.038) (Fig. 2). In contrast with that, no correlation in homocysteine levels has been found between HF patients with MA and MOA (*p* = 0.25 and *p* = 0.99, respectively).

As predictable from the literature, there was a statistically significant difference between homocysteine levels and the polymorphism of MTHFR: patients with T/T genotype have significant higher homocysteine levels than C/C genotype and C/T genotype (*p* < 0.0001 and *p* < 0.0001, respectively) with differences between C/C genotype and C/T genotype (*p* = 0.65).This result is consistent in both male (*p* = 0.03) and female (*p* = 0.008) individuals.

## Discussion

Several studies over the last decade have investigated the relationship between the MTHFR C677T genotype and susceptibility to migraine. Previous literature in pediatric populations has been conflicting and limited by small study size (*n* = 16 to *n* = 45) [4, 5, 8, 9, 12] (Table 2). One retrospective study of 41 patients with migraine reported a similar association between the T/T genotype and migraine to our results but included only 11 patients who had a TT genotype; 5 had MA and 5 MOA [8]. Our study is therefore the first to systematically investigate the relationship in a large pediatric population.

We performed MTHFR C677T polymorphism research for both diagnostic and therapeutic purposes in our migraine patients, aiming to reduce both frequency and severity of migraine attacks by dietary supplementation with folic acid in patients with high homocysteine blood levels, based on previous literature reports in children and adults [4–9, 12].

The limitations of using a retrospective study meant that there is no control group of patients without headache that have MTHFR C677T genotyping. So we compared the headache patients with patients referring to our clinic for other problems not related to headache, who did the MTHFR C677T genotyping for other reasons [4–7, 12].

There is also a risk of recall bias in patients and their parents’ self-reporting age of onset of headache symptoms, which may have influenced the results.

The C677T allele of MTHFR results in low levels of circulating folate, lower availability of methionine and high concentrations of homocysteine, which would increase the risk of endothelial vascular damage mediated by oxidative stress [3, 4, 7, 9]. It has been hypothesized that the resulting homocysteine-related endothelial dysfunction may be implicated in causing and perpetuating migraines. Application of the acid D, L-homocysteic, which mimics the effect of homocysteine, has been shown to increase the firing rate of trigeminal neurons responding to pain, further supporting the role of homocysteine in pain pathways and headache [3]. Moreover, some authors have suggested that MTHFR T/T mutations constitute a modest, but significant, genetic risk factor for ischemic stroke with conflicting results [8, 12].

The results of our retrospective study confirm the presence of a strong positive association between MTHFR T/T polymorphism and susceptibility to MA (up to 47.9% have the T/T polymorphism), in agreement with other results reported in pediatric and adult populations. In our study we also showed a significant, though smaller, association between the T/T genotype and MOA (up to 28.8% have T/T polymorphism), as already reported in other studies in non-Caucasian pediatric and adult populations [4, 6–9, 12, 13].

We also found a negative correlation between age of onset of migraine and T/T genotype. Our results suggest that the T/T genotype is related to earlier onset of migraine in a pediatric population (8.54 years compared with 8.89 years for C/T and C/C genotype) and in particular for MA (11.73 years compared with 12.50 in C/T e C/C genotype). This potential association has not previously been recognized and needs further confirmatory study.

Homocysteine levels were found to be significantly higher in patients suffering from MA (average value 7.85 μMol/L) compared to patients suffering from MOA (average value 5.82μMol/L). This result is in line with the comparatively higher frequencies of T/T genotype and suggests that the T/T genotype may affect homocysteine blood levels in children.

### By contrast, the study confirms the absence of correlation

Therefore, dietary supplementation with folic acid or a diet rich in folate to change homocysteine blood levels should be taken into consideration as an adjunct to other treatment in the long-term management of migraine and in particular in MA [4, 9].

Clearly, mutations in MTHFR alone are not the causal factor for migraine given that it is a complex, multifaceted disorder. However, considering the relationship between the C677T variant allele and MA is important to recognize the genetic and metabolic aspects of this condition. This would facilitate early treatment initiation in order to reduce the high levels of circulating homocysteine, improve the course of migraine and reduce longer-term risk of possible complications such as ischemic stroke.

## Conclusion

Our retrospective study performed in this large pediatric cohort of migraine patients provides evidence to support that MTHFR T/T homozygosity influences susceptibility to migraine and in particular in MA.

## Abbreviations

5, 10-CH2-THF: 5, 10- methylenetetrahydrofolate
5-CH3-THF: 5-methyltetrahydrofolate
bp: Base pair
HF: Headache Free
ICHD: International Classification of Headache Disorders
IHS: International Headache Society
MA: Migraine with aura
MOA: Migraine without aura
MTHFR: Methylenetetrahydrofolate reductase
PCR: Polymerase Chain Reaction
TTH: Tension-type headache
